# *Acanthamoeba* spp. genotypes demonstrate genotype-specific motility and encystment differences in both fed and starved environments

**DOI:** 10.3389/fopht.2025.1684686

**Published:** 2025-11-05

**Authors:** Allison Campolo, Esther Lara, Monica Crary

**Affiliations:** Microbiology, Alcon Research, LLC, Fort Worth, TX, United States

**Keywords:** *Acanthamoeba*, motility, encystment, keratitis, cornea

## Abstract

**Introduction:**

*Acanthamoeba* is a ubiquitous protozoan pathogen that can cause a severe ocular infection, *Acanthamoeba* keratitis. Despite its high prevalence and potential contamination of contact lenses, the natural behavior of this parasite remains poorly understood. Therefore, we investigated *Acanthamoeba* trophozoite movement, rate of encystment, trophozoite size, and phylogenetic relationships between eight prevalent *Acanthamoeba* genotypes.

**Methods:**

*Acanthamoeba* was seeded onto a plate with and without *E. coli*. After initial size measurements were recorded, images were taken using a microscope to create time-lapse videos over a 72-hour period. Amoeba trophozoite tracks were quantified for distance, displacement, and speed. Separately, *Acanthamoeba* cysts were generated naturally over the course of the study via nutrient deprivation in ¼ Ringer’s over 72 hours. Wells were stained with calcofluor white to identify cysts and wells were quantified for rate of encystment and cyst size.

**Results:**

Of the eight genotypes investigated, T7 and T18 possessed the largest trophozoite size while T5 was the smallest. T5 was consistently the fastest genotype over the 72-hour period in both the fed and starved conditions. Nutrient conditions did not show any consistent impact on the overall distance, speed, or encystment of any genotype within 72 hours. Finally, while some genotypes (T1, T11) demonstrated a relatively high percentage of encystment at the 24-, 48-, and 72-hour timepoints, the other genotypes demonstrated a relatively low encystment percentage at these same times.

**Discussion:**

Overall, these results indicate that eight of the common genotypes of *Acanthamoeba* vary widely in terms of size, speed, rates of encystment, and response to nutritional state. From these, we can infer that *Acanthamoeba* keratitis prevention methods must be robust enough to counter amoeba in trophozoite or cyst form, and that amoeba should be expected to be able to traverse a wide variety of distances (for instance, across a contact lens or onto a corneal epithelium) in either a fed or starved nutritional state.

## 1 Introduction

*Acanthamoeba* is a free-living amoeba commonly found in soil and water, such as tap water, swimming pools, and lakes, and is the cause of the devastating infection known as *Acanthamoeba* keratitis. For this reason, contact lens manufacturers and ocular health practitioners go to great lengths to educate society about the importance of contact lens hygiene (1–3). However, contact lens-wearer non-compliance remains extremely high (cited as 40 to 91 percent non-compliant) (4), thus putting contact lens-wearers at risk for developing *Acanthamoeba* keratitis. To combat this, it is critical that contact lens solutions are able to sufficiently overcome contact lens contamination events, which makes understanding the behavior of *Acanthamoeba* critically necessary.

Arguably, one of the most important behavioral aspects of *Acanthamoeba* is its ability to exist in either the trophozoite form, which is actively motile, or the cyst form, which is dormant and double-walled during times of stress. While trophozoite ambulation may represent a larger inherent danger as it can move across a contact lens or from a lens to a cornea, they have been shown to be susceptible to most contact lens solutions (5–9). Conversely, cysts, which are immobile, are extremely resistant to common biocides and must be specifically disinfected by either hydrogen peroxide or iodine povidone (9). However, the time it takes a trophozoite to encyst in either the presence or absence of nutrients is generally an under-investigated field of knowledge. It is generally believed that natural encystment can take days to weeks, but chemical induction of encystment through common ophthalmic viscosity agents like propylene glycol can reduce that induction to hours (10, 11). Delayed or inadequate decontamination of a contaminated contact lens significantly increases the likelihood of transferring amoebic cysts to the cornea (12).

Further, understanding these parameters – that is, amoeba motility and percentage and rate of encystment in timepoints relevant to contact lens wear - and how they correspond to many different genotypes of commonly encountered *Acanthamoeba* may significantly contribute to our understanding of the amoeba that is commonly seen in patients. Many papers have been written and studies conducted regarding the T4 genotype (13–15), and while that genotype is largely considered representative of infectious *Acanthamoeba* (15), it is crucial to expand investigations to other highly relevant genotypes that patients may encounter. We have included strains from genotypes T1, T2, T3, T5, T7, and T11 as representative genotypes that have less commonly been isolated from *Acanthamoeba* keratitis, and genotype T18 as a rare strain which has been isolated from granulomatous amoebic encephalitis and thus has the potential to be a human pathogen (16–19). Thus, we here explore strains representing eight genotypes in their encystment, as well as their size and motility. We have previously demonstrated that the T4 genotype (ATCC 30461 and ATCC 50370) possesses a remarkable ability to remain motile on various surfaces and in different nutritional states (5, 20). The current study illuminates the different capabilities of these eight genotypes of *Acanthamoeba* in their ability to maintain movement, as well as the speed at which a genotype may succumb to encystment in the absence of nutrients.

## 2 Materials and methods

### 2.1 *Acanthamoeba* culturing

As previously described (5, 20), an axenic culture medium (AC6 containing 20 g biosate peptone, 5 g glucose, 0.3 g KH_2_PO_4_, 10 ug vitamin B12, and 15 mg L-methionine per liter of distilled deionized water) was used to axenically produce *Acanthamoeba* trophozoites. Final pH was kept between 6.6-6.95 using 1 M NaOH and AC6 was then autoclaved at 121 °C for 20 minutes being stored at room temperature. Stored AC6 was used within three months of production. *Acanthamoeba* genotypes were purchased from the American Type Culture Collection (ATCC, Manassas, VA, USA). Genotypes used are described in **Table 1**.

**Table 1:** *Acanthamoeba* genotypes from American Type Tissue Collection (ATCC) used in this study with their genotype, scientific name, and isolation source.

Strain	Genotype	Name	Source
ATCC 50655	T1	*Acanthamoeba* sp. 13	Granulomatous Amoebic Encephalitis
ATCC 30872	T2	*Acanthamoeba polyphaga*	Freshwater
ATCC 50702	T3	*Acanthamoeba griffini*	Keratitis
ATCC 30010	T4	*Acanthamoeba castellani*	Soil
ATCC 50703	T5	*Acanthamoeba lenticulata*	Human Nose
ATCC 30137	T7	*Acanthamoeba astronyxis*	Soil
ATCC PRA-115	T11	*Acanthamoeba hatchetti*	Lens Case
ATCC PRA-411	T18	*Acanthamoeba byersi*	Granulomatous Amoebic Encephalitis

*Acanthamoeba* trophozoites were grown in fresh AC6 medium in the final 24 hours before use to ensure uniform proliferation and a homogenous population of trophozoites. Amoeba were collected and centrifuged at 500 x g for 5 minutes, after which the pellet was resuspended using ¼ Ringer’s solution. Count seeding was confirmed via manual counting using a hemocytometer. Amoeba were seeded into a 48-well plate at a concentration of 7.5x10^3^ cells/mL, with 1 mL per well. Half of the wells were also seeded with 10^6^ cells/mL *Escherichia coli* (ATCC 8739) to provide a nutrient source for the *Acanthamoeba*.

### 2.2 *Acanthamoeba* size and motility quantification

After seeding the wells, amoeba were allowed to settle and adhere to the plate for 20 minutes. Following confirmation that images produced similar size quantifications at 4X, 10X, and 20X magnifications, all wells were scanned for size at minute 20 at the 4X magnification using a 4-stitch image to capture the entire well for this study. Six replicates (i.e., six independent wells) were quantified for each genotype in order to calculate the average and standard error.

**Figure 1:**
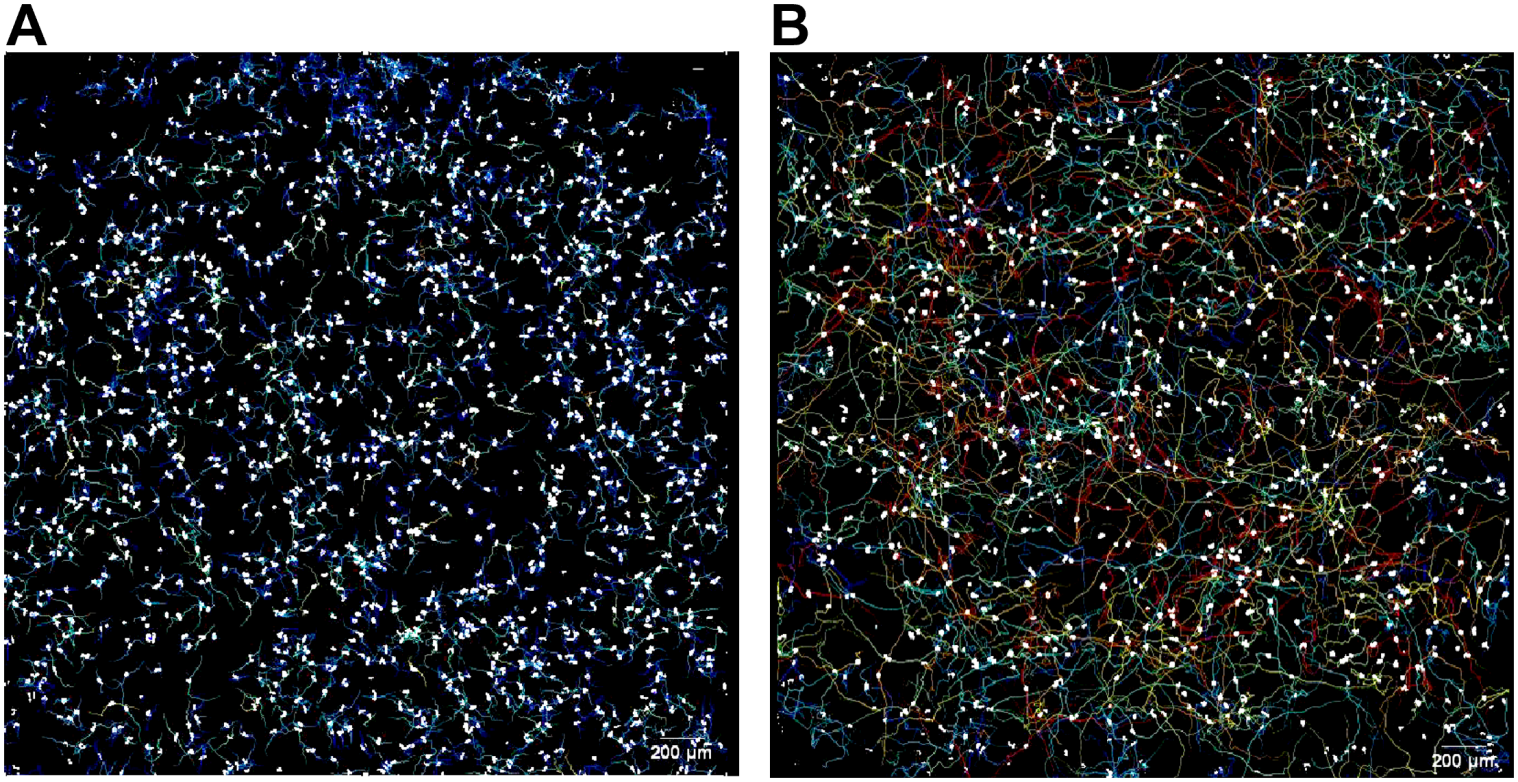
Representative image of motility analysis of **(A)** a relatively slower genotype observed in this study (T3, ATCC 50702), and **(B)** a relatively faster genotype observed in this study (T5, ATCC 50703), observed over 12 hours. Amoeba are shown as white circles on a black background, and the tracks they move along are shown as multi-colored lines. For more representative images and detailed methods of motility analysis please reference Campolo et al. (5) and Campolo et al. (21). Scale = 200µm.

Amoeba were tracked for 72 hours total. After sizing images were taken (i.e., at the 30-minute mark after cells were initially seeded), amoeba were imaged every 30 seconds at 4x magnification using a 2x1 stitched image of each well for hours 0.5-12, 24-25, 48-49, and 72-73 (**Figure 1**). As detailed previously (5, 20), amoeba tracking data was analyzed for total distance, maximum distance, and displacement, which are measured in microns traveled per timepoint (data gathered over 1 hour, 12 hours, or 24 hours) and speed (measured in microns per second). As a note, speed is calculated as microns per second per time period. Thus, as the time period become longer, the calculated speed becomes necessarily slower as divided over a longer time period.

### 2.3 *Acanthamoeba* cyst staining

*Acanthamoeba* cysts were generated naturally over the course of the study as amoeba were deprived of nutrients long term in ¼ Ringer’s for over 72 hours. At the 24-, 48-, and 72-hour time points, wells designated for each time point (each time point possessing independent sets of wells) were stained with Calcofluor White (Millipore Sigma, Darmstadt, Germany, Catalog #F1303), which binds to the cellulose of the cyst cell wall (22). Cells which stained blue under fluorescent microscopy were counted as cysts while cells which remained unstained were counted as trophozoites. The percentage of cysts in the population was determined by comparing cysts to the total cell count, and the encystment of each genotype at each time point was compared across genotypes at the same time point.

### 2.4 Phylogenetic tree

The evolutionary history was inferred by using the Maximum Likelihood method and Kimura 2-parameter model (23). Initial tree(s) for the heuristic search were obtained automatically by applying Neighbor-Join and BioNJ algorithms to a matrix of pairwise distances estimated using the Maximum Composite Likelihood (MCL) approach, and then selecting the topology with superior log likelihood value. There were a total of 3298 positions in the final dataset. Evolutionary analyses were conducted in MEGA11 (24).

### 2.5 Statistics

The data from each amoeba track within a replicate (i.e., each well) were averaged, and then replicates were averaged for a sample size of 3 for each time point, condition, and genotype. Statistical analysis was completed via two-way repeat measures ANOVA for comparison between genotypes within each condition and time point, and within each genotype’s own baseline. This comparison was followed by Tukey’s *post hoc* multiple comparisons test and significance was set at *p* < 0.05. Due to the high number of comparisons per graph and to maintain legibility of each graph, *p*-values for between-condition comparisons in the quantification of movement between the eight genotypes are displayed in the **Supplementary Table 1**. We further calculated the effect sizes of key genotypes and time points using Cohen’s d, and this graph is in the **Supplementary Figure 1** as well.

## 3 Results

**Figure 2:**
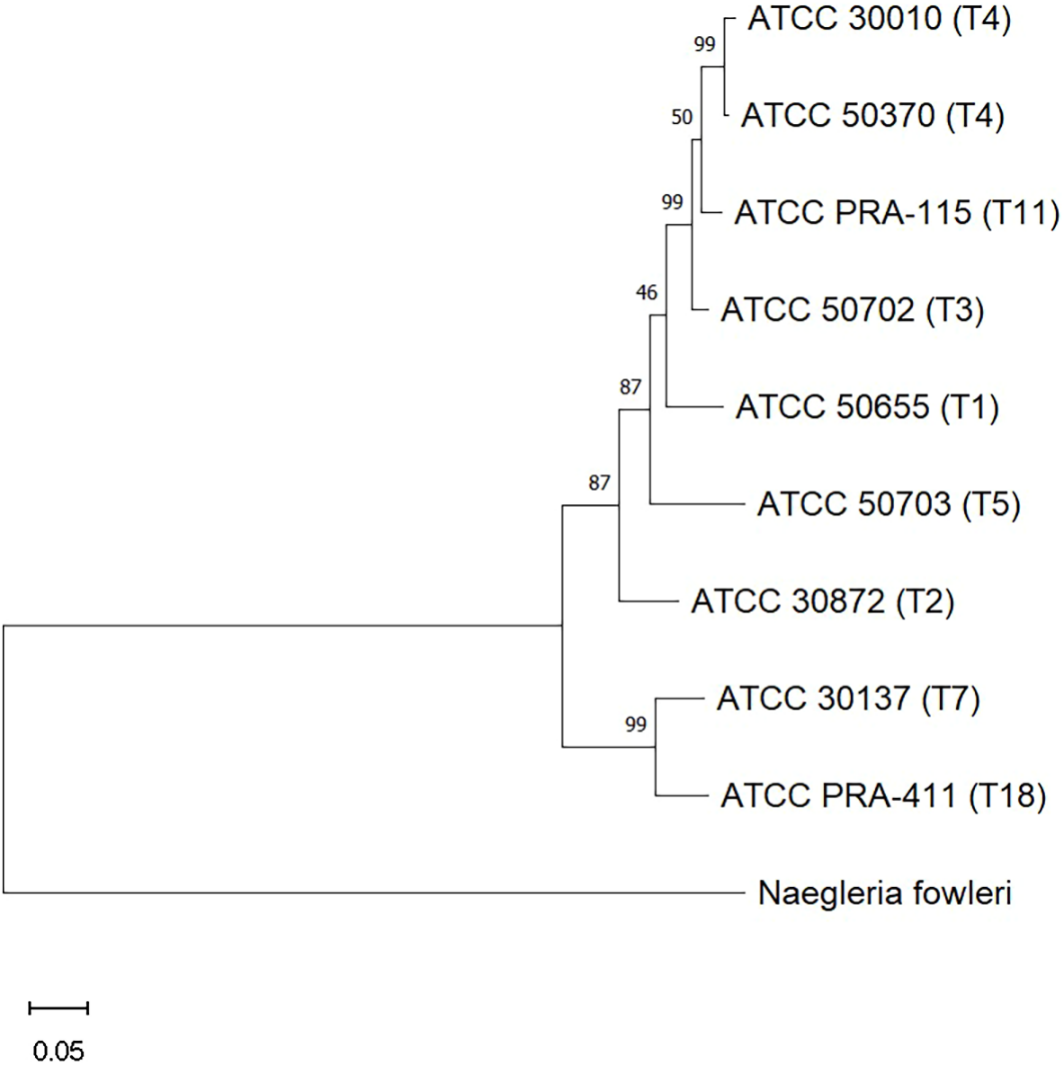
Evolutionary analysis of relationships between the *Acanthamoeba* genotypes used in this study, as determined by the Maximum Likelihood method. Genotypes are identified by their ATCC numbers, followed by their genotype in parentheses. Measures of support are given in percent at each branch. Scale bar = 0.05; the distance scale represents the number of nucleotide substitutions per site between sequences.

In this study we chose to examine eight different genotypes of *Acanthamoeba*, several of which have been infrequently associated with *Acanthamoeba* keratitis research. While we and others have often published data regarding ATCC 30461 and ATCC 50370 from genotype T4 (13–15), this study examined ATCC 50655, 30872, 50702, 30010, 50703, 30137, PRA-115, and PRA-411 from genotypes T1, T2, T3, T4, T5, T7, T11, T18, respectively. These genotypes were found to be free of endosymbionts to prevent confounding variables of the impact of diverse endosymbionts on *Acanthamoeba* biology. The phylogenetic relationship between these genotypes (**Figure 2**) demonstrates the diversity seen within the genus, *Acanthamoeba*, and equally lends credence to the differences being observed in their behavior.

**Figure 3:**
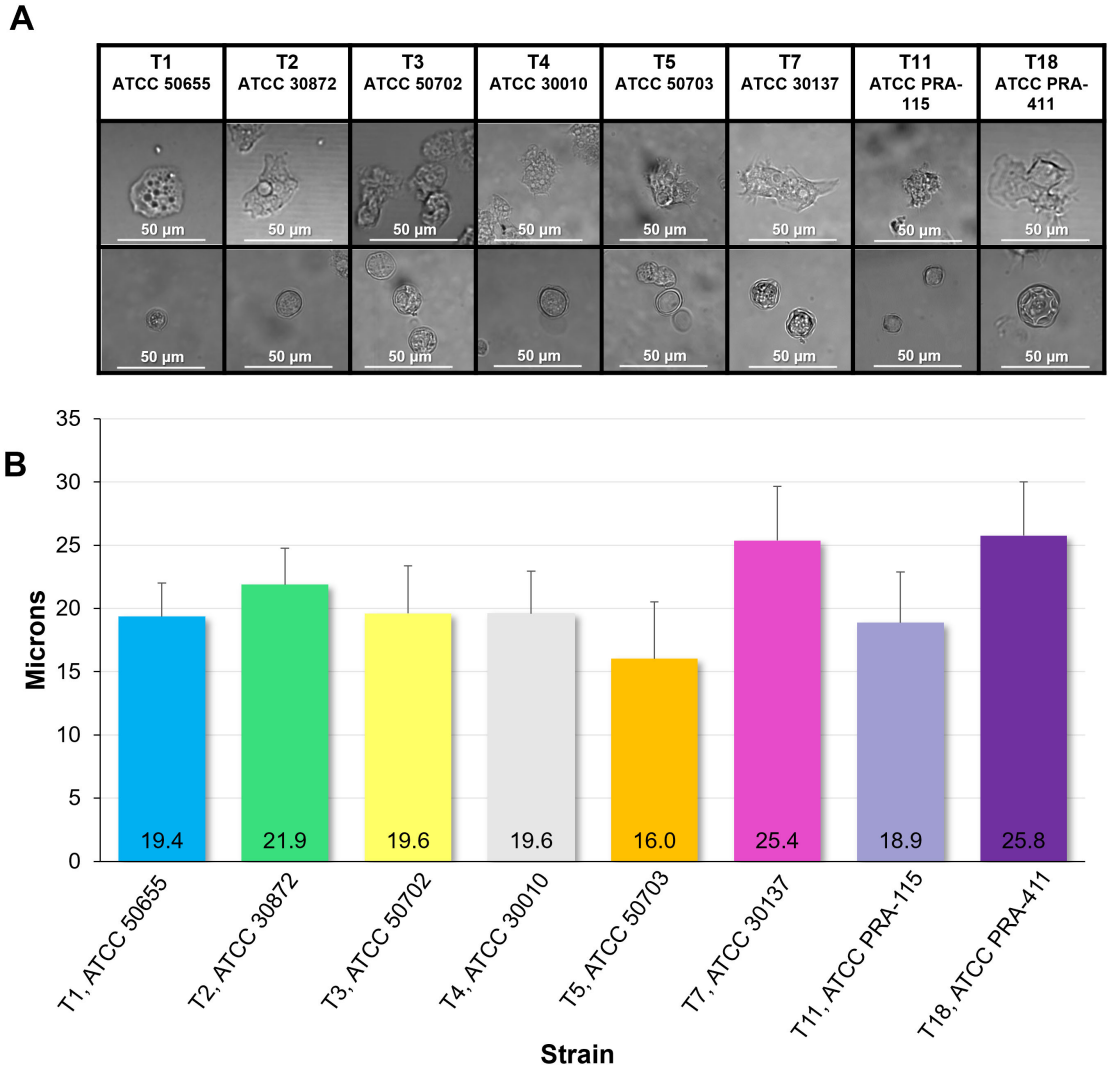
Morphology and size of the eight genotypes investigated. **(A)** Representative images of genotype morphology divided by trophozoites (top row) and cysts (bottom row). **(B)** Mean ± SE size of trophozoites divided by genotype. n=6 sample wells per genotype, each with an average quantified size of trophozoites in the well of 7.5 x 10^3^ cells in a 1mL suspension.

All eight genotypes were visually examined at 40X magnification to understand the morphology of these genotypes in both their trophozoite and cyst forms (**Figure 3**). Trophozoites were measured for size, and the means of the sizes of each genotype were compared. T7 and T18 were the two largest genotypes with an average diameter of over 25µm while T5 was the smallest genotype with an average trophozoite diameter of 16µm. Most of the genotypes fell easily within the known spectrum of previously studied *Acanthamoeba* specimens in that they are between 12 and 50 µm (17).

**Figure 4:**
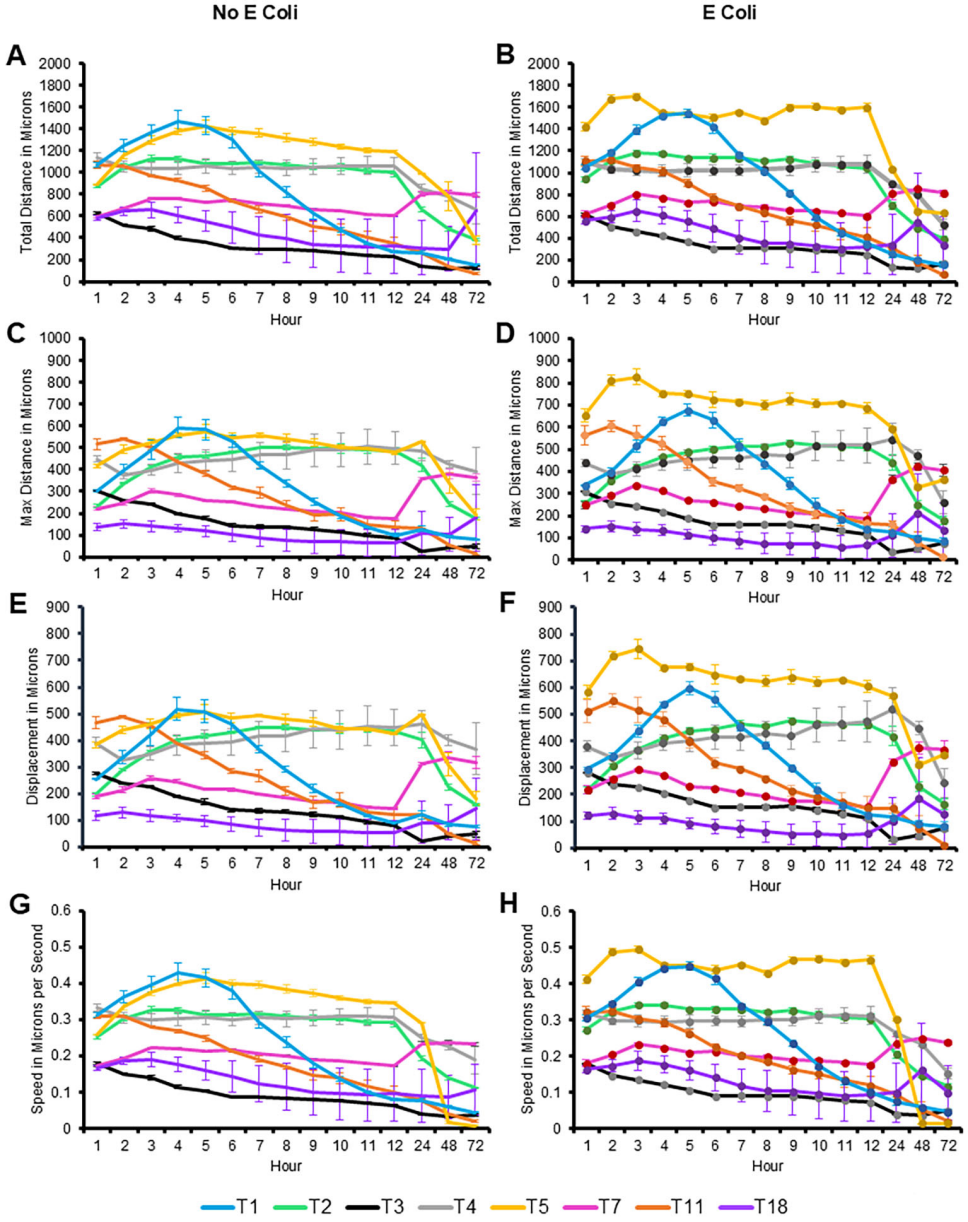
Quantification of movement of the eight genotypes investigated over 72 hours, with and without *E. coli*. Mean ± SE of **(A)** total distance, **(B)** total distance with *E. coli*, **(C)** max distance, **(D)** max distance with *E. coli*, **(E)** displacement, **(F)** displacement with *E. coli*, **(G)** speed, **(H)** speed with *E. coli*. Statistical comparisons presented in **Supplementary Table 1**. n=3 replicates per group/time point/genotype/*E. coli* status, with 150–650 individual tracks per replicate.

All eight genotypes were monitored over 72 hours for motility via measuring total distance (in microns), max distance (the straight distance between the start point and the furthest point the amoeba travelled at any time), displacement (the straight distance between the start point and the end point to determine if the amoeba travels in a relatively straight line or more of a circular pattern), and speed in microns per hour (**Figure 4**). These measurements were taken in either the absence or presence of nutrients (heat-killed *E. coli*). In both states, T5 was the genotype that consistently moved the furthest, and the fastest from hours 1 through 48, with the only obvious decrease being at hour 72. T5 showed a significant increase in movement and speed particularly in the presence of *E. coli*.

In contrast, T3 and T18 were consistently the slowest genotypes examined, with the exception being T18’s increase in distance travelled at hour 72, in both the fed and starved conditions. T1 was the only genotype to show an increase in motility, peaking between hour 4–5 before decreasing steadily across the course of the experiment. Most genotypes maintained their motility with only slight reductions until the 24-hour timepoint. The other genotypes did not exhibit significant differences in their motility patterns between the fed and starved conditions, indicating a relatively stable rate of movement regardless of nutrient availability. This suggests that the presence of *E. coli* did not have a substantial impact on all genotypes. While motility was not closely related to phylogenetic relationships, similar patterns between T3/T11 and T7/T18 were observed.

**Figure 5:**
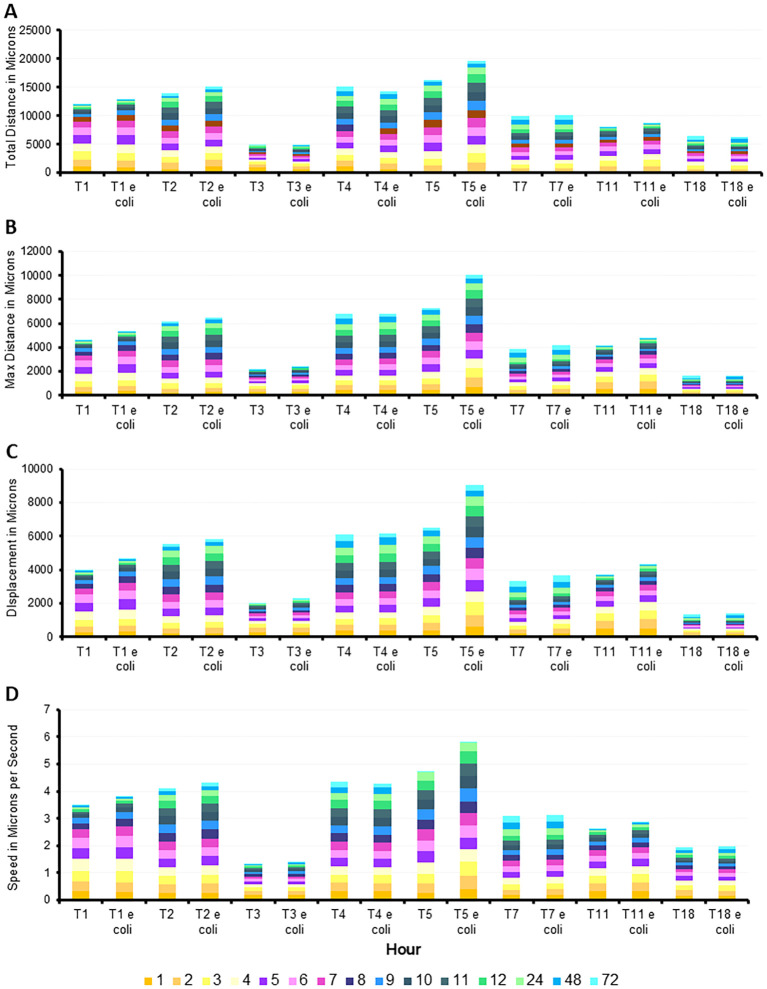
Additive quantification of motility averages by genotype, with and without *E. coli*, with hours identified by color. Summation of **(A)** total distance, **(B)** max distance, **(C)** displace, and **(D)** speed. Averages of each quantification determined from n=3 replicates per group/time point/genotype/*E. coli* status, with 150–650 individual tracks per replicate.

The statistical comparisons for **Figure 4** are provided in the **Supplementary Tables**. This same data was alternatively presented by quantifying the number of microns each genotype moved per hour. This allowed for clearer comparison of distance and speed travelled between genotypes under fed and starved conditions (**Figure 5**). It is similarly evident that T3 was consistently the slowest genotype while T5, particularly in the fed condition, was the fastest genotype.

The detailed analysis of the movement patterns reveal that T5’s motility is significantly enhanced in the presence of *E. coli*, suggesting a strong positive response and a unique sensitivity to nutrient availability. Conversely, T3 and T18 indicate a potential delayed response to environmental trophic status. These findings highlight the variability in motility among different genotypes and underscore the potentially limited influence of nutrient presence on amoeba movement, depending on genotype.

**Figure 6:**
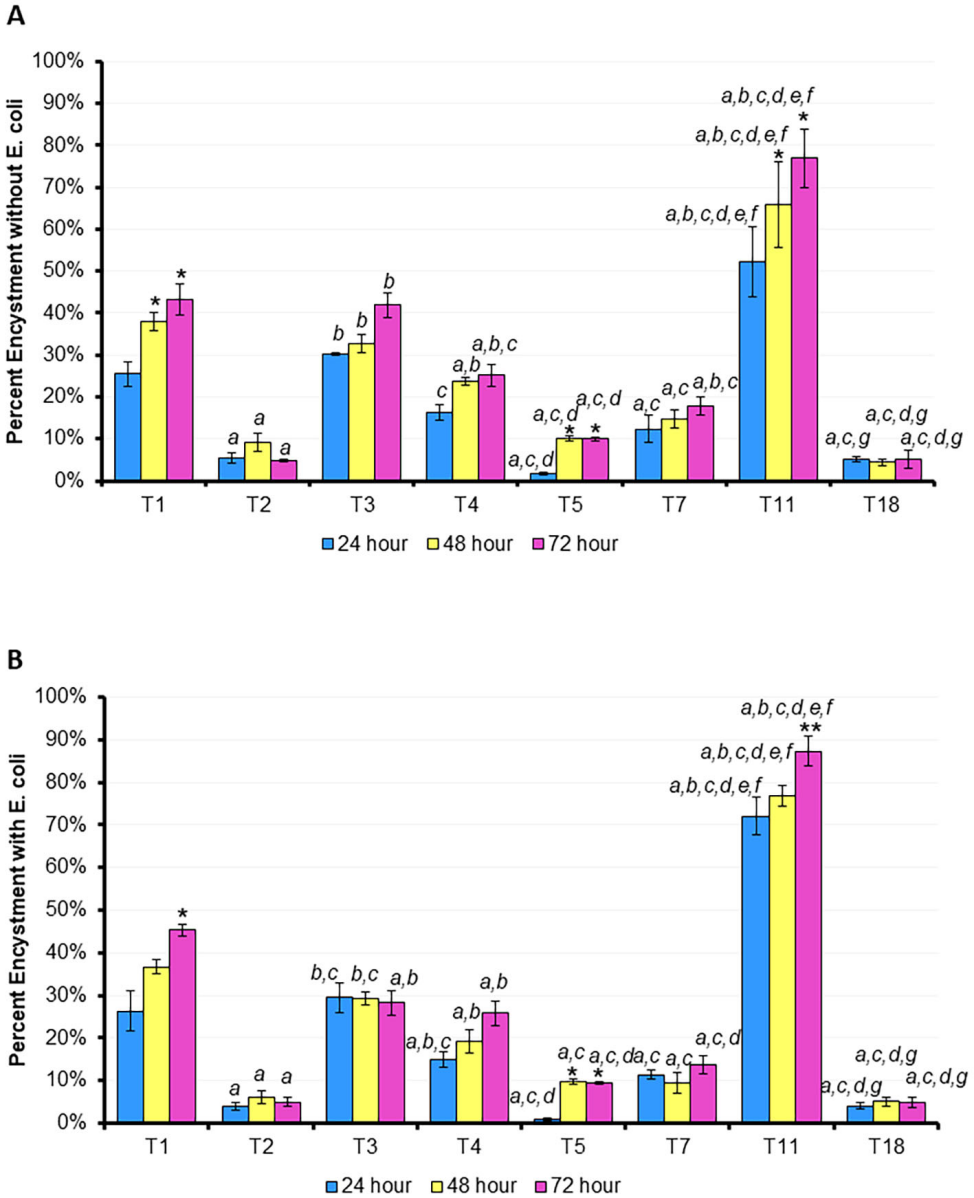
Percentage of trophozoites which have encysted after 24, 48, or 72 hours. **(A)** Mean ± SE percentage encystment without *E. coli*, **(B)** mean ± SE percentage encystment with *E. coli*. n=3 per genotype/time point. Statistical comparisons at the same time point between genotypes: *a* p < 0.05 vs. T1 (ATCC 50655), *b* p < 0.05 vs. T2 (ATCC 30872), *c* p < 0.05 vs. T3 (ATCC 50702), *d* p < 0.05 vs. T4 (ATCC 30010), *e* p < 0.05 vs. T5 (ATCC 50703), *f* p < 0.05 vs. T7 (ATCC 30137), *g* p < 0.05 vs. T11 (ATCC PRA-115); T18 is ATCC PRA-411; within one genotype between time points: * vs. 24 hours, ** vs. 48 hours; via 2-way RM ANOVA with *post hoc* Tukey’s test.

Finally, by staining the cells with calcofluor white at 24, 48, and 72 hours, we were able to measure the percent encystment of each genotype examined in both fed and starved conditions (**Figure 6**). With the exception of T1 and T11, the genotypes investigated did not demonstrate a significant difference in encystment between the timepoints. T1 and T11 demonstrated significantly more encystment at hours 48 and 72 than hour 24 in the starved state, and more encystment at hour 72 than hour 24 in the fed condition. T11 demonstrated the highest rate of encystment at all time points in both the fed and starved conditions while T2, T5, and T18 demonstrated the least encystment in both conditions.

## 4 Discussion

*Acanthamoeba* is a genus of free-living amoeba found in diverse environments such as soil, water, and air. These amoebae exhibit remarkable motility and long-term survival techniques, which play a crucial role in their nutritional needs and the pathogenesis of *Acanthamoeba* keratitis. In this study, we compared the motility characteristics and rate of encystment of eight different prominent *Acanthamoeba* genotypes which are not often examined in the context of ocular health (25).

We recognize that *Acanthamoeba* maintain a unique ability to harbor endosymbionts, which can add a substantial variable to any *Acanthamoeba-*related investigation. Thus, to reduce variability, we chose *Acanthamoeba* without endosymbionts (as indicated by a lack of reports regarding these strains as containing internal contaminants, and as confirmed in our lab via repeated culturing without antibiotics without any contamination events) to ensure that *Acanthamoeba* biology was not being influenced by the internal interactions with any bacteria.

Different *Acanthamoeba* genotypes can exhibit varying pathogenic potential (26). Understanding the phylogenetic relationship between the different genotypes can help inform the clinical community on any particular pathogenesis. Therefore, we examined the phylogenetic relationships between the eight genotypes examined here (27). We found the closest relationships between T3 and T11. Further, we determined that T1, T2, T3, T4, T5, and T11 are on a separate clade from T7 and T18. These relationships are evident in the motility and encystment data gathered here: closely related genotypes may have similar rates of speed and encystment, but the overall variety of responses demonstrate the diversity in *Acanthamoeba* biology and its adaptability to its own environment. Future analyses may reveal that these relationships are further apparent in contact lens care disinfection assays, revealing genotype-to-genotype similarity in susceptibility to biocides, but this remains to be investigated.

In this manuscript we also compared the sizes of *Acanthamoeba* trophozoites by taking detailed images of fresh amoeba populations. The sizes of our trophozoites and cysts are in line with the available published data, in which trophozoites are generally cited as being 12 to 50µm in diameter, and cysts are slightly smaller at 10 to 25µm in diameter (17). Among the eight genotypes we examined, T7 and T18 possessed the largest trophozoites, both with average diameters of 25µm. The smallest genotype was T5, at 16µm. As expected, representative images confirm that within each genotype, the cyst form is smaller in diameter than the trophozoite form.

Interestingly, among these eight genotypes, trophozoite size appears to have little to no relationship to motility or rate of encystment. For instance, T7, T18, and T5, despite have the biggest differences in size between them, have very similar rates of encystment. T5 is the smallest trophozoite of this group, has one of the lowest rates of encystment, and is also consistently the fastest at the most timepoints, but the next smallest trophozoite, T11, is consistently at the middle of the pack in terms of motility and has highest percentage of encystment. In this same vein, T11 has the significantly largest percentage of encystment both with and without *E. coli* present, but again is not the slowest or fastest trophozoite, nor does it have a substantially different size than the other genotypes.

Except for T5, all eight genotypes maintained some amount of movement through the 72-hour period. T5 was often the fastest genotype both with and without *E. coli*, but reduced its speed dramatically after 24 hours compared to the other genotypes. All seven other genotypes maintained some level of movement in both the fasted and fed conditions, including the next-fastest genotypes, T1 and T2, and the slowest genotypes, T18 and T3. We hypothesized that providing a heat-killed *E. coli* nutrient broth would allow amoeba to move further and faster, and to prevent encystment, but this was not the result we observed. While there was a slight increase in motility in the fed state for T5, all seven other genotypes demonstrated essentially no difference in motility between the two conditions over the 72 hours period. Similarly, T11 demonstrated a modest decrease in encystment at each timepoint in the fed state versus the starved one, but the other seven genotypes demonstrated essentially no alteration in encystment between the two conditions. T2, T5, T7, and T18 demonstrated the least encystment (<20%) in both the fed and fasted states while T1, T3, and T4 demonstrated a moderate amount (20-50%) of encystment in both states. Notably, our T4 encystment rates are in line with those published by other groups using similar experimental conditions (28, 29). T11, T1, and T5 were also the only genotypes to demonstrate a significant difference in encystment compared to itself: hours 48 and/or 72 were significantly different from hour 24 within this genotype. It is interesting that these three strains also encysted at such high levels in the presence of available nutrients. The drive to encyst in this scenario seems to be independent of starvation, leading to questions about the potential trigger. The T11 genotype in particular encysts at a much higher rate than all 7 other genotypes at all time points, which may be aid in its long term survival even if it limits the ability to find nutrients and proliferate in the trophozoite form. In contrast, the other five genotypes demonstrated no inner-genotype difference between time points. The speed and trigger of encystment may be an evolutionary advantage to avoid harsh environments or alternately to not initiate a terminal process like encystment unnecessarily. This makes the genus *Acanthamoeba* particularly challenging to study, as even closely related genotypes can exhibit highly complex and variable responses.

In conclusion, eight clinically important but often under-reported genotypes were examined here for phylogeny, motility, and encystment, with and without *E. coli* as a food source. We determined that all eight genotypes maintained motility for 24 hours, and seven of them maintained motility for at least 72 hours. All genotypes examined in this study possessed trophozoites which were between 16 and 26 microns in diameter. When comparing each time point, most genotypes did not demonstrate a significant difference in encystment. The presence of *E. coli* did not produce large or numerous differences between motility or encystment in most genotypes.

While this study definitively expands our understanding of *Acanthamoeba* motility and encystment, this examination is not without its limitations. To start, while we expanded the study of *Acanthamoeba* genotypes beyond what is often reported, we acknowledge that there are 23 *Acanthamoeba* genotypes and our study cannot represent them all. We also recognize that encystment can potentially take anywhere from a few days to weeks, and while we aimed to take this into account with a prolonged 72-hour time point, it is possible that a longer study held out for many days could provide further insights. Further, while our study was relatively large considering the number of time points, genotypes, and conditions, it may be enhanced in the future by including more replicates per group. Finally while heat-killed *E. coli* is a standard nutrient source for *Acanthamoeba* in the laboratory setting due to it being a controlled variable and *E. coli* a common pathogen contaminating contact lenses which can in turn be used as an amoebic food source, we recognize that this nutrient delivery method lacks the complexity of real-world scenarios.

These data indicate that *Acanthamoeba* continues to be a significant threat to ocular health and that amoeba can continue to possess the motility to colonize a contact lens or a cornea even in the absence of nutrients. Similarly, the rate of encystment – and thus the further challenges to disinfection – will be similar both with and without *E. coli*. The noted differences between the genotypes examined here lead to the conclusion that future studies quantifying the disinfection efficacy of contact lens solutions should consider expanding the number of genotypes used for such studies. Altogether, these findings emphasize the need for a more nuanced understanding of genotype-specific behavior in *Acanthamoeba*, which may ultimately give rise to the development of more effective diagnostic tools, treatment strategies, and disinfection protocols tailored to the diverse biology of this resilient organism.

## Data Availability

The original contributions presented in the study are included in the article/**Supplementary Material**. Further inquiries can be directed to the corresponding author.
