# Supplementary material for: Acanthamoeba spp. genotypes demonstrate genotype-specific motility and encystment differences in both fed and starved environments
Source: Front Ophthalmol (Lausanne). 2025 Nov 5;5:1684686. doi: 10.3389/fopht.2025.1684686 (PMC12626805; doi:10.3389/fopht.2025.1684686)
Supplement: Supplementary file 1 [file DataSheet1.docx]

Supplementary Material

# Supplementary Data

Supplementary Material should be uploaded separately on submission. Please include any supplementary data, figures and/or tables. All supplementary files are deposited to FigShare for permanent storage and receive a DOI.

Supplementary material is not typeset so please ensure that all information is clearly presented, the appropriate caption is included in the file and not in the manuscript, and that the style conforms to the rest of the article. To avoid discrepancies between the published article and the supplementary material, please do not add the title, author list, affiliations or correspondence in the supplementary files.

# Supplementary Figures and Tables

**
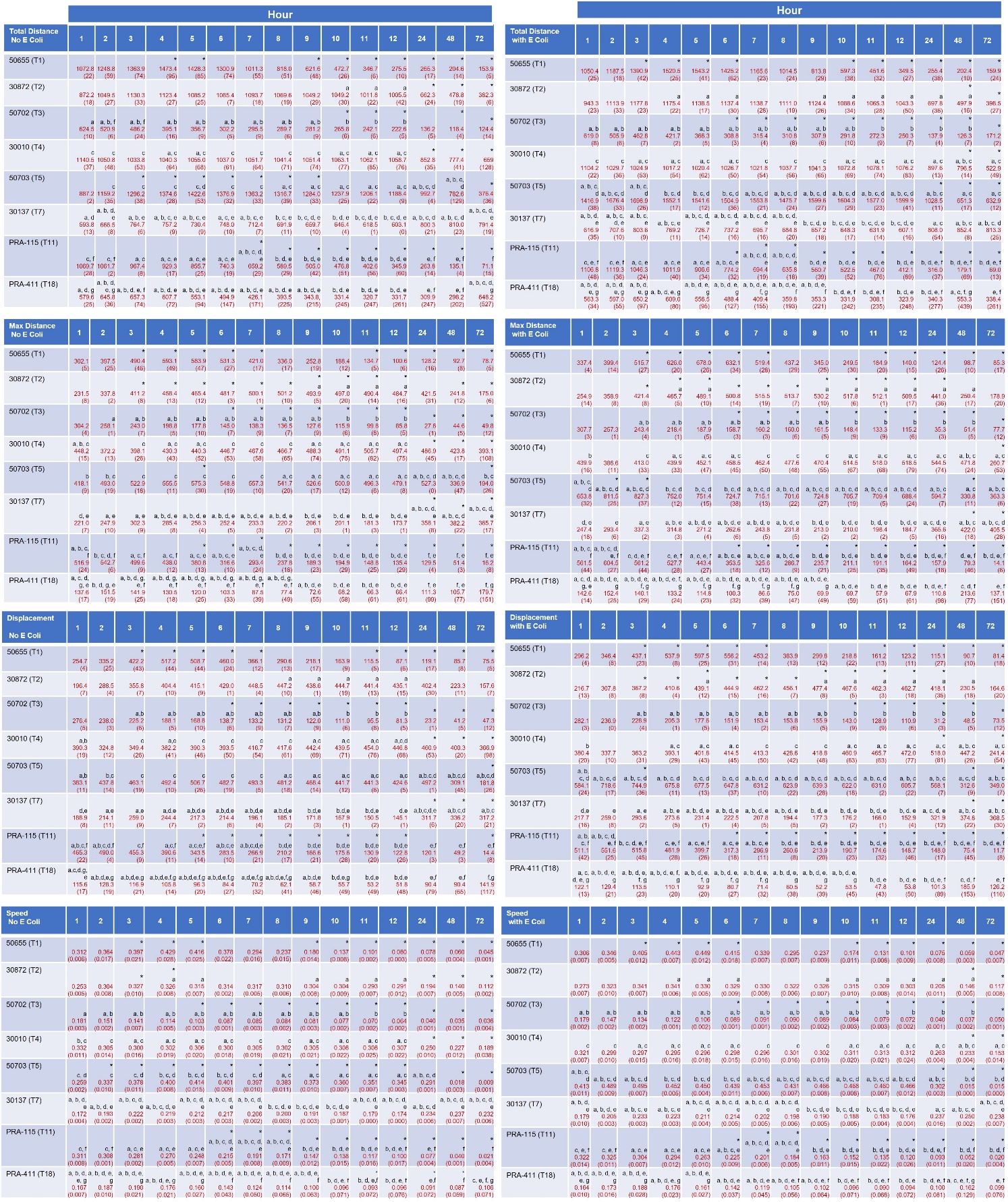
Supplementary Table 1.** Statistical comparisons for Figure 3 between strains. ***a*** p < 0.05 vs. T1 (ATCC 50655), ***b*** p < 0.05 vs. T2 (ATCC 30872), ***c*** p < 0.05 vs. T3 (ATCC 50702), ***d*** p < 0.05 vs. T4 (ATCC 30010), ***e*** p < 0.05 vs. T5 (ATCC 50703), ***f*** p < 0.05 vs. T7 (ATCC 30137), ***g*** p < 0.05 vs. T11 (ATCC PRA-115); T18 is ATCC PRA-411; within one genotype between time points: ***** vs. 1 hour; via 2-way RM ANOVA with post hoc Tukey’s test.


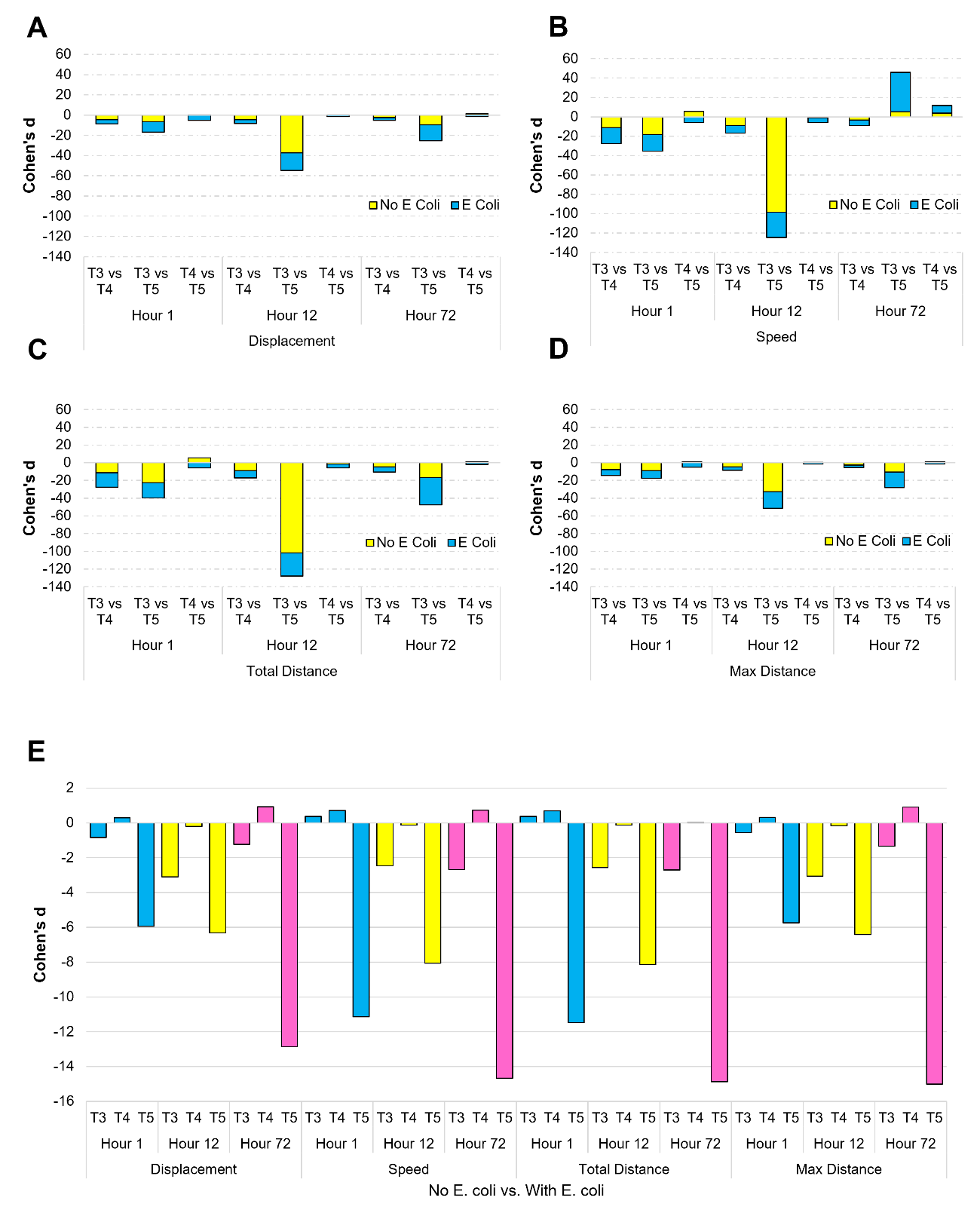


**Supplementary Figure 1.** Effect sizes between key genotypes (T3 being the relatively slowest genotype examined in this manuscript, T4 being more average and also the most-often reported on in the literature, and T5 being the relatively fastest genotype) at key time points (hour 1, hour 12, and hour 72) calculated using Cohen’s d. Effect sizes are shown for **A)** Displacement, **B)** Speed, **C)** Total Distance, **D)** Max Distance, and **E)** the difference within each genotype at each time point between amoeba with and without *E. coli* available as a nutrient source. **Note:** the scale bar on the y axis for subpanel E is smaller than for the other for subpanels due to the relatively much less difference between the fed and fasted states observed in this study.
